# Angiomyolipome rénal épithélioïde malin (AREM): à propos d’un cas rare

**DOI:** 10.11604/pamj.2019.33.64.11971

**Published:** 2019-05-28

**Authors:** Abdelouahed Lasri, Mohammed Alae Touzani, Mounir Lahyani, Tarik Karmouni, Khalid Elkhader, Abdellatif Koutani, Ahmed Ibn Attya Andaloussi

**Affiliations:** 1 Service d’Urologie B, CHU Ibn Sina, Faculté de Médecine et de Pharmacie, Université Mohammed V Souissi, Rabat, Maroc

**Keywords:** Angiomyolipome épithélioïde malin, marqueurs musculaires lisses et mélanocytaires, la néphrectomie totale élargie, Malignant epithelioid angiomyolipoma, smooth muscle cell markers and melanocytic markers, complete radical nephrectomy

## Abstract

L’angiomyolipome rénal épithélioïde malin (AREM) est une entité pathologique rare longtemps considérée comme lésion hamartomateuse et dont le diagnostic positif est purement immuno-histochimique, microscopiquement, il se caractérise par des cellules mononuclées épithélioïdes a cytoplasme clair présentant des atypies cytonucléaires sévères, et exprimant les marqueurs musculaires lisses et mélanocytaires (HMB 45). Nous rapportons un cas rare d’AREM diagnostiqué après analyse anatomopathologique d’une tumeur retropéritonéale dont l’origine ne pourrait être précisée préalablement par la tomodensitométrie.

## Abstract (en)

Malignant renal epithelioid angiomyolipoma (EAML) is a rare disease which has long been considered a hamartomatous lesion. Positive diagnosis is purely based on immunohistochemical investigation. Microscopically, it is characterized by mononuclear epithelioid cells showing a clear cytoplasm with severe cytonuclear atypies and expressing smooth muscle cell markers and melanocytic markers (HMB 45). We here report a rare case of EAML diagnosed after anatomopathological examination of a retroperitoneal tumor whose origin wasn’t predetermined by CT scan.

## Introduction

Angiomyolipome (AML) est une tumeur rénale bénigne commune composée des vaisseaux sanguins à paroi épaisse, de muscle lisse et de tissu adipeux, mais la variante épithélioïde maligne est extrêmement rare. Nous rapportons un cas d’angiomyolipome rénal épithélioïde malin (AREM) chez une patiente de 32 ans, diagnostiqué après néphrectomie et étude anatomopathologique de la pièce.

## Patient et observation

Il s’agit d’une patiente de 32 ans, sans antécédents médico-chirurgicaux particuliers, qui présente des lombalgies droites sans notion de fièvre ou d’hématurie, évoluant depuis 15 jours. La patiente ne décrit pas de facteurs déclenchant. L’examen clinique est peu contributif, retrouve un état général conservé, un abdomen souple avec une sensibilité du flanc droit, les aires ganglionnaires sont libres.

**Figure 1:**
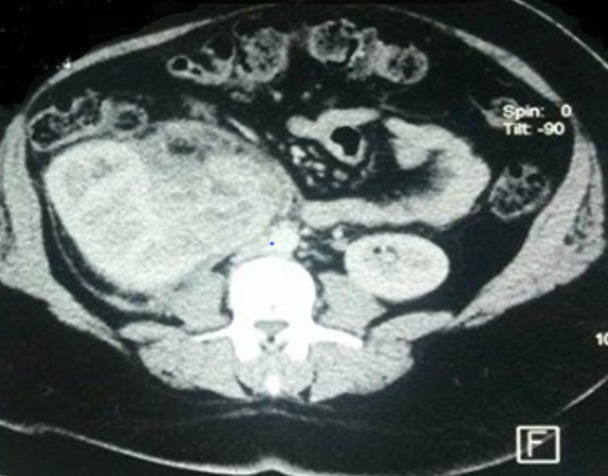
Coupe axiale TDM montrant un processus rénal polaire inferieur, refoulant les organes de voisinage

L’exploration biologique n’a pas objectivé de syndrome inflammatoire ou infectieux (vitesse de sédimentation: 6 mm/h, protéine C réactive inférieure à 5 mg/l, procalcitonine à 0,06 ng/ml), la numération formule sanguine est la fonction rénale sont normaux. L’échographie rénale a montré un rein droit augmenté de volume, arrivant jusqu’au pelvis, siège d’un processus hypoéchogène, hétérogène mesurant 10cm environ. Le complément scannographique confirme l’origine rénale polaire inférieure, et décrit une masse hétérogène à double composante liquidienne et tissulaire, se rehaussant de façon hétérogène après injection du Polycarbonates (PC); s’étendant en sous péritonéal et refoulant les structures avoisinante (VCI, ovaire droit, caecum), la graisse péri rénale parait infiltrée (Figure 1). Une néphrectomie totale élargie a été réalisée, macroscopiquement la masse est de couleur brune jaunâtre, friable, mesurée à 13,5 × 9,5 × 8,5cm de taille, à la coupe elle renferme des foyers nécrotique et hémorragiques.

**Figure 2:**
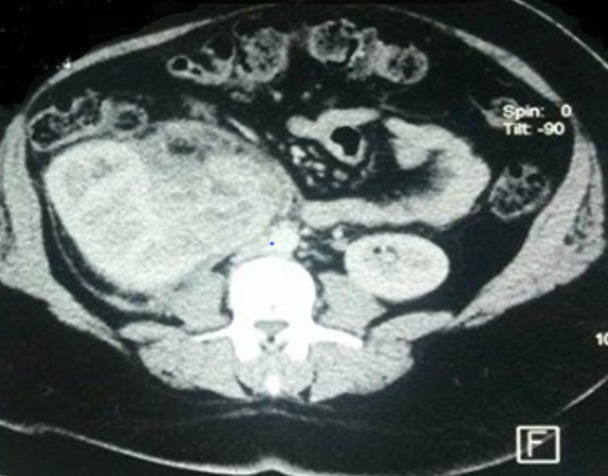
A) cellules rondes et polyédriques épithélioïdes à noyaux polymorphes; B) vaisseaux à paroi épaisse au contact des cellules épithélioïdes

**Figure 3:**
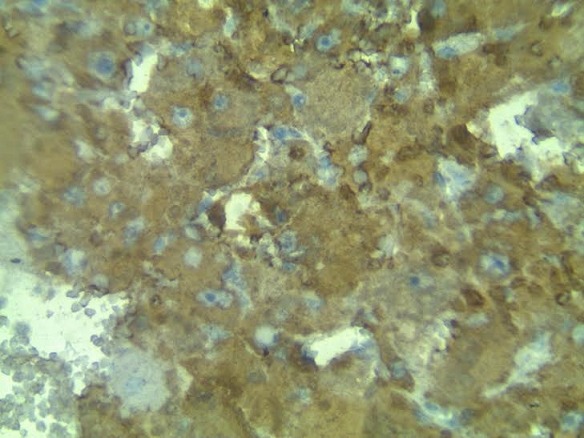
Marquage des cellules épithélioïdes avec l’anticorps anti-HMB45

Au microscope, la tumeur présente de volumineuses cellules épithélioïdes polymorphes, dotées de noyaux ronds de grandes tailles, parfois poly-nucléées, avec un cytoplasme éosinophile abondant, des dépôts d’hémosidérine et de cholestérol sont observés ainsi que des foyers de nécrose tumorale. Les figures de mitoses sont estimées à 3 mitoses par 10 CFG, La tumeur associe un contingent vasculaire fait de vaisseaux à parois dystrophiques (Figure 2). L’immunomarquage cytoplasmique montre une forte expression de l’HMB45 et du Melan-A (Figure 3). Le contrôle à un mois retrouve une patiente en bonne état général, le scanner est sans anomalies.

## Discussion

L’angiomyolipome est la tumeur conjonctive la plus fréquente du rein. Cette tumeur peut être sporadique ou se développer dans le cadre d’une sclérose tubéreuse de Bourneville (STB) [1]. Les angiomyolipomes épithélioïdes rénaux (AMLeR) sont des tumeurs rares (identifiées chez moins de 0,1 patients pour 1000 habitants) et représentent 8% des angiomyolipomes (AML) opérés. L’AMLeR est une masse rénale solide qui atteint principalement les femmes (jusqu’à 78%). L’âge moyen de découverte est de 40 ans avec des extrêmes de 14 à 70 ans [1-3].

Les AML épithélioïdes peuvent se rencontrer également dans le foie, le rétropé-ritoine, les ganglions, l’utérus ou l’os. Ils appartiennent à la famille des pecomes pour perivascular epithelioid cells [1-4]. La cellule mésenchymateuse rénale périvasculaire se multiplie et se différencie en tumeur bénigne, majoritairement triphasique: cellules musculaires lisses, adipocytes et vaisseaux dystrophiques. Les AML sont positifs en immunohistochimie pour HMB-45 et Melan-A qui sont situés sur les mélanosomes [5]. La particularité des AML épithélioïdes est d’avoir, par rapport à la forme classique, un potentiel d’évolutivité de récidive ou de métastase.

Les AMLeR peuvent être sporadiques ou associés à la STB (de 31 à 50%) [5, 6]. Cette association est plus fréquente que l’association des AML classiques avec la STB (31% vs 20%) [6]. Les AMLeR sporadiques sont plus souvent symptomatiques que les cas associés à la STB [6]. Les AMLeR sporadiques bénins regroupent des formes uniques et des formes multiples. Le diagnostic des AMLeR sporadiques bénins est théoriquement rétrospectif, caractérisé par l’absence d’évolution locorégionale ou à distance [6]. Pouvant être découverts suite à des douleurs (50%), une masse palpable dans le flanc (10%), une hématurie (20%), une hypertension artérielle (HTA) ou une fièvre [7, 8].

Les tumeurs de petite taille sont asymptomatiques dans trois cas sur quatre, mais au-delà de 4cm, plus de la moitié des AML devient symptomatiques, nécessitant une prise en charge. La vitesse de croissance n’est pas prévisible justifiant la surveillance semestrielle [8-10]. Les AMLeR sporadiques bénins ne provoque pas d’altération de l’état général (AEG) et leur évolution est lente [6]. Les AMLeR sporadiques bénins uniques sont les formes les plus fréquentes [6]. Les AMLeR bénins multiples peuvent représenter 30% des AMLeR [6]. Les localisations d’AMLeR sporadiques bénins multiples peuvent être rénales uni- ou bilatérales et même ganglionnaires (lymphangioleiomyomatose (LAM)) et extrarénales [5].

Les AMLeR à potentiel agressif et malins regroupent les formes cliniques avec AEG, ou à évolution locale rapide, les formes avec extension à la graisse périrénale, avec envahissement vasculaire et avec thrombus cave [11, 12], les formes avec extension ganglionnaire associée et les formes avec des métastases à distance. Des localisations ganglionnaires avec une histologie d’AMLeR de mauvais pronostic sont des métastases ganglionnaires (diagnostic différentiel avec la LAM) [5]. Les AMLeR malins avec métastases à distance ont des localisations hépatiques, pulmonaires, osseuses, neurologiques, spléniques, péritonéales ou testiculaires. Les métastases d’AMLeR malins ont parfois un comportement agressif [6].

Il est difficile de différencier AML épithélioïde malin d’autres tumeurs rénales solides tels un oncocytome, un carcinome à cellules rénales, ou un sarcome et par les techniques d´imagerie seule. Le scanner et l’imagerie par résonnance magnétique sont les plus utilisés pour détecter les foyers de graisse dont la présence et massive et caractéristique dans les AML classiques.

Cependant, certains aspects radiologiques peuvent suggérer le diagnostic, incluant une densité supérieure au parenchyme rénal normal sur les scanner non injecté, des contours du rein déformés, pauvre en densité graisseuse, avec un rehaussement nettement hétérogène après injection du PC (rapidwash-in and slow wash-out), de rares zones de nécrose et occasionnellement des métastases ganglionnaires sans invasion du sinus rénal [13]. Le diagnostic d’AMLeR est histologique sur pièce de néphrectomie totale, partielle ou sur biopsie à l’aiguille fine. Dans l’AMLeR, il y a un contingent de cellules épithélioïdes et périvasculaires. Le critère qualitatif est retenu mais certaines études utilisent un critère quantitatif, 5% de la tumeur prélevée [14] ou en majorité [2]. Les AMLeR sont souvent développés au dépend de forme triphasique. Les AMLeR peuvent être confondus avec des sarcomes de haut grade. Les critères d’agressivité histologique de l’AMLeR sont: une anaplasie nucléaire, une activité mitotique élevée (> 1/50 High Power Field (HPF)), une invasion vasculaire, la présence de nécrose et une infiltration de la graisse péri rénale [4-15]. Les marqueurs de différenciation mélanocytaires comme HMB-45 ou Melan-A en immunohistochimie sont positifs dans tous les AML [2, 14]. Les AMLeR font partie des tumeurs à cellules épithélioïdes périvasculaires [2 , 14, 15]. Parmi les formes malignes de PEComes rapportées, les AMLeR malins rénaux sont les plus fréquents [15]. L’AMLeR malin avec envahissement locorégional est défini par un envahissement locorégional et une AEG ou une évolution rapide, un aspect de masse rénale indéterminée à la tomodensitométrie (TDM), une tumeur supérieure à 5cm, un contingent infiltrant, un haut grade nucléaire, de la nécrose ou une activité mitotique supérieure à 1/50 HPF sont souvent associé.

La néphrectomie totale élargie devrait être indiquée pour les AMLeR malins avec envahissement locorégional [16]. Un traitement adjuvant par doxorubicine doit être discuté pour les AMLeR malins avec envahissement locorégional [11, 12]. La néphrectomie totale élargie devrait être indiquée pour les AMLeR malins avec métastases à distance. Le curage ganglionnaire des AMLeR malins avec métastases à distance est indiqué si les ganglions sont visibles au TDM ou peropératoire. Un traitement adjuvant par doxorubicine semble efficace pour les AMLeR malins avec métastases à distance. Un traitement adjuvant par rapamycine peut être discuté pour les AMLeR malins avec métastases à distances. La survie des patients atteints d’AMLeR malins varie considérablement, la néphrectomie totale élargie semble donner de bon résultats dans les formes localisés, cependant le pronostic est péjoratif dans les maladies métastasiques malgré le traitement adjuvante [17].

## Conclusion

L’AMLeR malin est une variante des AML classiques, ayant un caractère agressif, difficile à distinguer du CCR en préopératoire, d’où l'intérêt d’un bon examen anatomo-pathologique permettant de confirmer le diagnostic.

## Conflits d’intérêts

Les auteurs ne déclarent aucun conflit d'intérêts.
